# Supplementary material for: Content-rich biological network constructed by mining PubMed abstracts
Source: BMC Bioinformatics. 2004 Oct 8;5:147. doi: 10.1186/1471-2105-5-147 (PMC528731; doi:10.1186/1471-2105-5-147)
Supplement: Additional File 2 — The original results of the above study (non-essential files are deleted to keep the file size under the limit set by BMC bioinformatics). [file 1471-2105-5-147-S2.bz2 › chilibotAdditionalFile2/dip20/224ID7831290E740/html/PLCG1_SYK.html]

 


 **PLCG1** and **SYK** 
  
Found 77 abstracts in PubMed, retrieved 20.  
 

 What does Google say? 
 PDF only 
| .edu only 

---

**Interactive relationship** (e.g. stimulation, inhibition, etc)

**Stimulatory relationship**- We also show that Tec family PTKs enhanced endogenous phospholipase C PLC gamma1  [ **PLCG1** ]  phosphorylation induced by CD8 zeta and  **Syk**  in 293T cells.  Ref: 11240141 FEBS Lett, 2001
- Activation of src and  **Syk**  kinases plays a significant role in signal transduction of heterophil degranulation probably by stimulating downstream phosphorylation of PLC  [ **PLCG1** ] , PLD, and PI3 K.  Ref: 12757738 Int Immunopharmacol, 2003
**Neutral relationship**- FcepsilonRI ligation is able to induce major signaling events like protein tyrosine kinase activation including p72  **syk**  leading to PLC gamma1  [ **PLCG1** ]  phosphorylation and consecutive calcium influx.  Ref: 11306920 Int Arch Allergy Immunol< MedlineTA>Int Arch Allergy Immunol, 2003
- H 2 O 2 induced p38 activation is abrogated in phospholipase C gamma2 PLC  [ **PLCG1** ]  gamma2 deficient as well as  **Syk**  deficient cells, suggesting that  **Syk**  activates p38 through PLC  [ **PLCG1** ]  gamma2 upon H 2 O 2 stimulation.  Ref: 12215219 Antioxid Redox Signal, 2002
- Finally, in cells deficient in the PLC  [ **PLCG1** ]  gamma2, one of the phosphorylated substrates regulated by  **Syk**  and Btk, TPA induced PLD activation, as well as phosphatidylinositol bisphosphate PIP2 hydrolysis was remarkably reduced.  Ref: 11380624 Genes Cells, 2001

**Non-interactive relationship** (e.g. studied together, co-existance, homology, etc.)

- Inhibition of the tyrosine kinase  **Syk**  by piceatannol does not affect vWF induced tyrosine phosphorylation of Fc gamma RIIA but prevents phosphorylation of PLC  [ **PLCG1** ]  gamma 2.  Ref: 11344169 J Biol Chem, 2001
- In B cells from Cbl b deficient mice cross linking the BCRs resulted in sustained phosphorylation of Igalpha,  **Syk** , and phospholipase C PLC  [ **PLCG1** ]  gamma2, leading to prolonged Ca2 mobilization, and increases in extracellular signal regulated kinase ERK and c Jun NH2 terminal protein kinase JNK phosphorylation and surface expression of the activation marker, CD69.  Ref: 12771181 J Exp Med< MedlineTA>J Exp Med, 2001
- Fc varepsilon RI induced tyrosine phosphorylation of phospholipase C gamma PLC  [ **PLCG1** ]  gamma but not  **Syk**  was significantly suppressed in these cells.  Ref: 12810085 Biochem Biophys Res Commun, 2003
- Coligation of the cytoplasmic region of PD 1 with the B cell receptor BCR in IIA1.6 transformants inhibited BCR mediated growth retardation, Ca 2 mobilization, and tyrosine phosphorylation of effector molecules, including Ig beta,  **Syk** , phospholipase C gamma 2 PLC  [ **PLCG1** ]  gamma 2, and ERK1 2.  Ref: 11698646 Proc Natl Acad Sci U S A, 2001
- These findings have led to the suggestion that BLNK is required for coupling  **Syk**  to PLC  [ **PLCG1** ]  gamma2, thereby accelerating cell apoptosis in B cells exposed to low doses of H2O2.  Ref: 11813980 Antioxid Redox Signal, 2001
- The Src family kinase  **Syk** , the B cell adaptor protein BLNK, and phospholipase PLC  [ **PLCG1** ]  gamma2 were consistently absent from Reed Sternberg cells.  Ref: 12881301 BloodBlood, 2001
- CONCLUSIONS We demonstrated that the  **Syk** , Btk and PLC  [ **PLCG1** ]  gamma2 pathways are required for TPA induced PLD activation in DT40 cells.  Ref: 11380624 Genes Cells, 2001
- Endogenous B cell receptors BCR coupled through  **Syk**  kinase to phospholipase C gamma PLC  [ **PLCG1** ]  gamma activated the expressed TRPC3 channels in both DT40w t and DT40InsP 3 R k o cells.  Ref: 11466302 J Biol Chem, 2001
- Calcium mobilization in BLNK deficient as well as  **Syk**  deficient and phospholipase C PLC  [ **PLCG1** ]  gamma2 deficient cells after H2O2 treatment was completely abolished.  Ref: 11813980 Antioxid Redox Signal, 2001
- WGA, which is a very potent platelet stimulator, elicited a rapid surge in  **Syk**  and phospholipase C PLC  [ **PLCG1** ]  gamma 2 tyrosine phosphorylation and the resultant intracellular Ca 2 mobilization.  Ref: 11669637 Biochemistry, 2001
- In contrast, the tumor cells in all cases of lymphocyte predominance Hodgkin rad s disease were positive for Fyn,  **Syk** , BLNK and PLC  [ **PLCG1** ]  gamma2, and Lyn immunostaining was seen in a minority of biopsies.  Ref: 12881301 BloodBlood, 2001
- Engaging CRs on chicken heterophils activates a proximal tyrosine kinase src and  **Syk**  kinases >PLC  [ **PLCG1** ]  PLD >PI3 K >JNK signal transduction pathway that induces degranulation.  Ref: 12757738 Int Immunopharmacol, 2003
- Similarly, vWF induced tyrosine phosphorylation of Fc gamma RIIA, as well as of  **Syk**  and PLC  [ **PLCG1** ]  gamma 2, occurs normally in aspirinized platelets.  Ref: 11344169 J Biol Chem, 2001
- Engaging Fc receptors on chicken heterophils activates a  **Syk**  >PLC  [ **PLCG1** ]  >PI3 K >p38 MAPK signal transduction pathway that induces degranulation.  Ref: 12188037 Int Immunopharmacol, 2002
